# Time course of complications after small renal mass biopsy: evaluation of initial follow-up images

**DOI:** 10.1007/s11604-023-01509-9

**Published:** 2023-11-22

**Authors:** Soichiro Kajita, Toshihiro Iguchi, Yusuke Matsui, Koji Tomita, Mayu Uka, Noriyuki Umakoshi, Takahiro Kawabata, Kazuaki Munetomo, Takao Hiraki

**Affiliations:** 1 Department of Radiology, Okayama University Hospital, 2-5-1 Shikata-Cho Kita-Ku, Okayama, 700-8558 Japan; 2 Department of Radiology, Okayama Saidaiji Hospital, Okayama, Japan; 3 Department of Radiological Technology, Faculty of Health Sciences, Okayama University, Okayama, Japan; 4 Department of Radiology, Faculty of Medicine, Dentistry and Pharmaceutical Sciences, Okayama University, Okayama, Japan

**Keywords:** Biopsy, Imaging, Complication, Renal neoplasms

## Abstract

**Purpose:**

To retrospectively assess the time course of complications after image-guided small renal mass biopsy using initial follow-up imaging.

**Materials and methods:**

A total of 190 masses (mean, 2.1 ± 0.70 cm; range, 0.6–3.8 cm) were assessed using initial computed tomography (43 non-enhanced and 141 enhanced) or magnetic resonance imaging (five non-enhanced and one enhanced) after biopsy. Initial follow-up imaging was classified into two groups (i.e., with or without hematoma) and various factors were compared.

**Results:**

The masses were histologically diagnosed in all patients except one. Post-procedural complications included 129 Grade I hematomas, 1 Grade I hemothorax, 9 Grade II hematomas, and 1 Grade IIIa pneumothorax. Residual 28 Grade I and 6 Grade II hematomas and 8 new complications (6 small hematomas, 1 pseudoaneurysm, and 1 arteriovenous fistula) were observed on the initial follow-up imaging obtained at a median of 21 days (3–90 days) after the biopsy. On the initial follow-up imaging, the groups with and without hematoma differed significantly in the following factors: age (*P* = 0.04), size (*P* = 0.02), guided images (*P* < 0.01), hematoma at the end of the procedure (*P* < 0.01), and days after biopsy (*P* < 0.01). Although three masses exhibited > 25% shrinkage, no significant change was observed in mass diameter on initial follow-up imaging (mean, 2.1 ± 0.71 cm; *P* = 0.90).

**Conclusion:**

Initial follow-up imaging after a biopsy revealed improvements in most of the complications, a few new complications, and an unchanged mass diameter.

## Introduction

Approximately 20% of small renal masses (i.e., ≤ 4 cm) are benign [1], and it is crucial to avoid unnecessary surgical resection and local ablative therapy (e.g., cryoablation and radiofrequency ablation). When small renal masses are difficult to diagnose using imaging studies before surgery or when local ablative therapy is planned, an image-guided needle biopsy is performed for histological evaluation [2, 3].

Image-guided needle biopsy for renal masses was previously contraindicated due to its low diagnostic performance and the risk of some complications (e.g., bleeding and seeding) [4]. Conversely, recently this procedure has been frequently performed with high diagnostic yields and safety; > 90% of renal masses can be diagnosed histologically [5]. Because complications are often minor and asymptomatic in image-guided needle biopsy, no imaging follow-up is usually performed (e.g., follow-up computed tomography [CT] is performed 1 week or 1 month after biopsy). We speculated that follow-up imaging studies would show changes in the biopsy-related early complications as well as additional findings (e.g., occurrence of late complications and increase in mass diameter). However, few robust and large reports exist on post-biopsy imaging studies.

This study aimed to retrospectively assess the time course of complications on initial follow-up CT or magnetic resonance imaging (MRI) after renal mass biopsy.

## Materials and methods

This study was approved by our ethics committee (approval number: KEN2202-039) and opt-out consent was obtained for the retrospective use of medical patient data. This study was conducted according to the principles of the Declaration of Helsinki. Informed consent was waived because of the retrospective use of patient data; however, written informed consent was obtained from all patients before they underwent percutaneous renal mass biopsy and imaging studies.

## Patients

This study included patients who underwent percutaneous image-guided cutting needle biopsy of renal masses at our institution between April 2016 and August 2020. The inclusion criteria were as follows: (i) ≤ 4 cm renal mass (i.e., T1a renal cell carcinoma [RCC] equivalent); and (ii) after the biopsy, initial follow-up CT or MRI was conducted in the period between the next day to 90 days. The exclusion criteria were as follows: (i) patients in whom biopsy was performed for multiple renal masses during the study period; (ii) initial follow-up CT or MRI was performed after another abdominal interventional radiology procedure (e.g., transarterial embolization [TAE] and biopsy of another abdominal mass); (iii) masses for which re-biopsy after a non-diagnostic initial biopsy was performed; (iv) masses for which biopsy was performed after TAE in the same session; or (v) biopsy performed for masses with local progression after renal cryoablation.

## Biopsy procedure

A biopsy was performed by 14 interventional radiologists with a median experience of 17 years (range, 5–32 years), using a coaxial system with an 18- or 20-gauge cutting needle (TEMNO Evolution, MERIT MEDICAL, UT, USA or STARCUT, TSK LABORATORY, Tochigi, Japan). Patients who were taking anticoagulants or antiplatelet medications underwent biopsy procedures either after discontinuation of these medications or after conversion to heparinization.

After confirming the target and setting the puncture route, all biopsies except one were performed either under CT fluoroscopy or ultrasound (US) guidance under local anesthesia. One biopsy was performed under the general anesthesia. Specimen acquisition was repeated until a sufficient sample was obtained for histological evaluation. After biopsy completion, complications were evaluated by US or plain conventional CT examination according to the Clavien–Dindo classification [6].

## Follow-up imaging and angiography

**Fig. 1:**
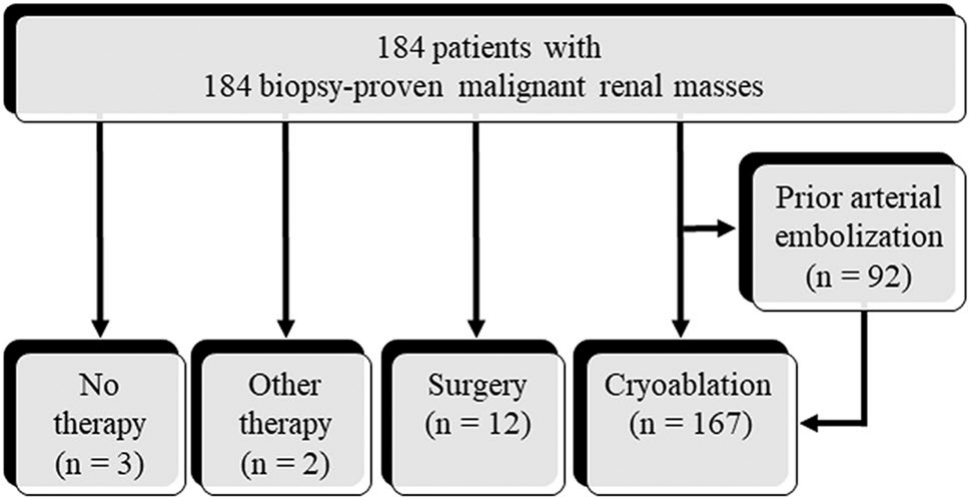
Sequence of events after a diagnosis of malignancy

The initial follow-up CT or MRI after the biopsy was performed in the period between the next day to 90 days. The sequence of events after a diagnosis of malignancy is shown in Fig. 1. In many patients with biopsy-proven RCC, angiography and TAE using lipiodol were performed before CT fluoroscopy-guided cryoablation to enhance tumor localization and reduce the risk of bleeding and seeding. First, a 4-F vascular sheath was placed via the common femoral artery. Next, digital subtraction angiography was performed after selecting the renal artery using a 4-F diagnostic catheter. In all TAE procedures, CT angiography was performed.

## Evaluation of the follow-up imaging results

Mass-related characteristics, such as size, laterality (right or left kidney), position (exophytic, parenchymal, mixed, or central), longitudinal location (upper or lower), and anteroposterior location (dorsal or ventral), were evaluated using CT or MRI performed before the biopsy. The positions and longitudinal and anteroposterior locations were categorized based on the definition by Gervais et al. [7] and Iguchi et al. [8], respectively.

The following findings were evaluated on CT or MRI: improvement in complications observed immediately after the biopsy, the occurrence of new complications not observed immediately after biopsy (i.e., delayed complications), and change in mass diameter. When angiography was performed, patients were evaluated for biopsy-related complications (e.g., occurrence of pseudoaneurysm and arteriovenous fistula [AVF]). If complications were detected, they were correlated with the initial follow-up CT or MRI findings. These images were reviewed by two board-certified diagnostic and interventional radiologists with 25 (T.I.) and 12 years (S.K.) of experience, respectively, by consensus.

## Statistical analysis

Initial follow-up images were classified into two groups, with or without hematoma. Patient-, mass-, and biopsy procedure-related factors were then compared between the two groups using Fisher’s exact test for categorical variables and the Mann–Whitney U test for numerical variables. The diameters of the renal masses on pre-biopsy and initial follow-up imaging were compared using paired t tests. Statistical significance was set at *P* < 0.05. All statistical analyses were performed using SPSS software version 26 (IBM).

## Results

**Fig. 2:**
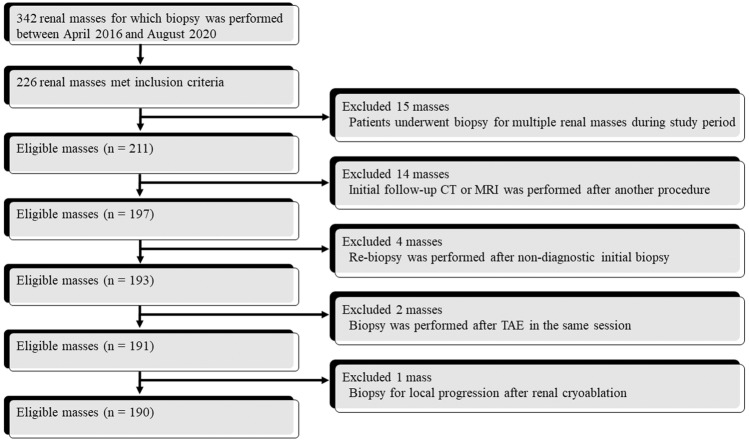
Flow chart diagram with the number of masses

An image-guided cutting needle biopsy was performed for 342 renal masses in 327 patients at our institution between April 2016 and August 2020. Among them, 190 masses (mean diameter, 2.1 ± 0.70 [standard deviation] cm; range, 0.6–3.8 cm) in 190 patients (129 men, 61 women; mean age, 64.8 ± 13.4 years; age range, 25–91 years) were included in this study (Fig. 2). The characteristics of the 190 patients, 190 masses, and 190 biopsy procedures are summarized in Table 1.

**Table 1:** Characteristics of 190 patients, 190 renal masses, and 190 biopsy procedures

Variable		Value
Patient		
Age (y)	Mean ± SD (range)	64.8 ± 13.4 (25–91)
Sex	Man/woman	129/61
Anticoagulants or antiplatelet medications	Yes/no	47/143
Initial follow-up image	Plain CT/Contrast-enhanced CT/Dynamic CT/Plain MRI/Dynamic MRI/CT angiography scans during TAE	43/7/98/5/1/36
Renal mass		
Size (cm)	Mean ± SD (range)	2.1 ± 0.70 (0.6–3.8)
Laterality	Right kidney/Left kidney/Renal allograft	102/86/2
Position	Exophytic/Parenchymal/Mixed/Central	140/10/22/18
Longitudinal location	Upper/Lower	99/91
Antero-posterior location	Dorsal/Ventral	105/85
Biopsy procedure		
Patient position	Prone/Supine/Lateral	183/2/5
Guiding image	Ultrasound/CT fluoroscopy (with contrast medium)/Both	42/140 (53)/8
Needle gauge	18-gauge/20-gauge/Both	186/3/1
Number of fires	Mean ± SD (range)	3.4 ± 1.1 (1–9)

SD: standard deviation, CT: computed tomography, MRI: magnetic resonance imaging, TAE: transarterial embolization

CT or US immediately after the biopsy showed 130 Grade I complications (129 hematomas and 1 hemothorax), 9 Grade II hematomas, and 1 Grade IIIa pneumothorax in 138 patients. In 189 masses (99.5%), a histological diagnosis was obtained revealing 184 malignancies and 5 benign lesions (Table 2). In one non-diagnostic biopsy, the histological finding was normal tissue (i.e., renal tissue with inflammation).

**Table 2:** Biopsy diagnosis of 190 renal masses

Histological diagnosis	Number
Malignancy	
Renal cell carcinoma	180
Metastasis	3
Unclassified carcinoma	1
Benign lesion	
Angiomyolipoma	1
Cyst	1
Oncocytoma	1
Papillary neoplasia	1
Renomedullary interstitial cell tumor	1
Non-diagnosis	
Renal tissue with inflammation	1

**Fig. 3:**
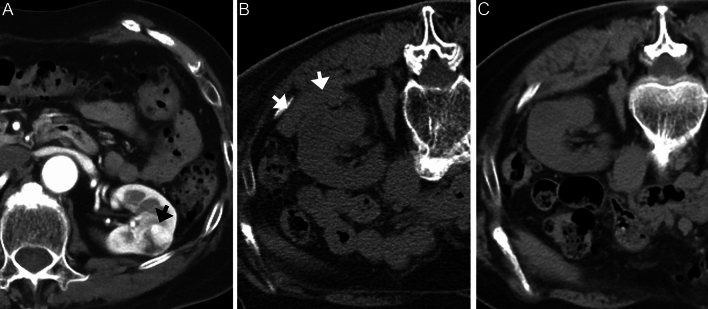
A 77-year-old woman with a renal mass in the left kidney. **A** Supine dynamic CT image (arterial phase) before biopsy shows a 2.1 cm renal mass (arrow). **B** Prone plain CT image immediately after US-guided needle biopsy shows a small hematoma (arrow). This mass is histologically diagnosed as clear cell RCC. **C** Prone plain CT image 25 days after biopsy shows disappearance of hematoma

Initial follow-up imaging was performed at a median of 21 days (3–90 days) after biopsy (Table 1). Fifty-six of 92 patients performed TAE already had initial CT or MRI before the TAE procedure. In the remaining 36 patients, initial follow-up CT was performed at the time of the TAE procedure. On the initial follow-up imaging, one hemothorax and one pneumothorax had disappeared after 21 and 33 days, respectively, and eight new complications (i.e., six small hematomas, one pseudoaneurysm, and one AVF) occurred. Of the 138 hematomas, 104 disappeared (Fig. 3), whereas 28 Grade I and 6 Grade II hematomas were reduced in size but remained. On the initial follow-up imaging, the groups with and without hematoma differed significantly in the following factors: age (*P* = 0.04), size (*P* = 0.02), guided images (*P* < 0.01), hematoma at the end of the procedure (*P* < 0.01), and days after biopsy (*P* < 0.01) (Table 3).

**Table 3:** Variables in presence and absence of hematoma on the initial follow-up image

			Hematoma		
Variable			Presence (n = 37)	Absence (n = 153)	*P* value
Patient					
	Age (y)	Mean ± SD (range)	69.3 ± 12.8 (44–91)	63.8 ± 13.2 (25–86)	0.04
	Sex	Man/Woman	28/9	101/52	0.33
	Post-biopsy anticoagulant or platelet medication	Yes/No	11/26	36/117	0.52
	Initial follow-up image days after biopsy	Mean ± SD (range)	13.1 ± 5.3 (3–23)	30.9 ± 18.6 (5–90)	< 0.01
Renal mass					
	Size (cm)	Mean ± SD (range)	2.3 ± 0.69 (1.1–3.6)	2.0 ± 0.69 (0.6–3.8)	0.02
	Laterality*	Right kidney/Left kidney	25/12	77/74	0.10
	Position	Exophytic and mixed/Others	34/3	128/25	0.30
	Longitudinal location	Upper/Lower	14/23	85/68	0.07
	Antero-posterior location	Dorsal/Ventral	25/12	80/73	0.10
Biopsy procedure					
	Guiding image**	Ultrasound/CT fluoroscopy	14/19	28/121	< 0.01
	Number of fires	Mean ± SD (range)	3.4 ± 1.2 (2–9)	3.4 ± 1.0 (1–8)	0.62
	Hematoma at the end of procedure	Presence/Absence	34/3	104/49	< 0.01
SD: standard deviation, CT: computed tomography, MRI: magnetic resonance imaging					

*Two renal masses in the renal allograft were excluded from the analysis

**Eight procedures performed under both image guidance were excluded from the analysis

**Fig. 4:**
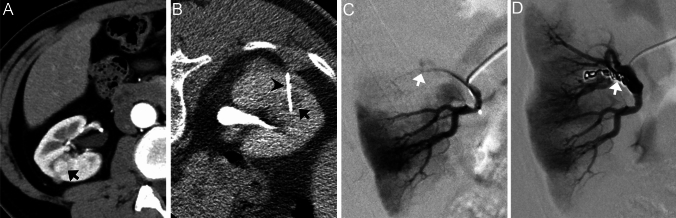
A 59-year-old man with a renal mass in the right kidney. **A** Supine dynamic CT image (arterial phase) before biopsy shows a 1.1 cm mass (arrow). **B** Prone CT fluoroscopy image with contrast medium during biopsy shows needle (arrowhead) penetration of the target mass (arrow). This mass is histologically diagnosed as clear cell RCC. **C** Selective renal angiography 78 days after biopsy shows AVF (arrow). **D** Selective renal angiography after coil embolization (arrow) shows the disappearance of AVF

After the biopsy, 167 RCCs were treated with cryoablation, and 92 underwent TAE before cryoablation. Of the 92 patients who underwent angiography, 1 had a pseudoaneurysm and AVF 13 days after the biopsy, and 2 had AVFs 31 and 78 days after the biopsy (Fig. 4). Of these complications, two AVFs could not be detected on the initial follow-up CT. All pseudoaneurysms and AVFs were successfully treated with TAE using the microcoils.

**Fig. 5:**
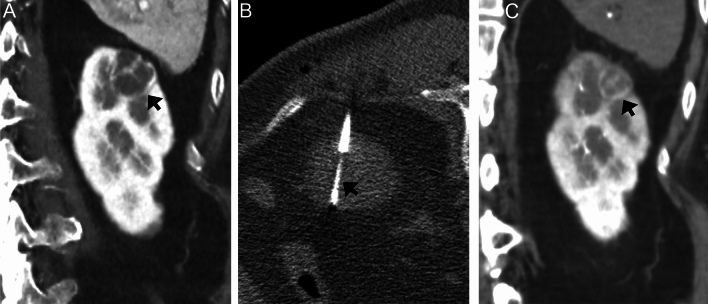
A 74-year-old woman with a cystic renal mass in the left kidney. **A** Coronal dynamic CT image (arterial phase) before biopsy shows a 2.3 cm mass (arrow). **B** Prone CT fluoroscopy image during biopsy shows needle (arrow) penetration of the mass. This mass is histologically diagnosed as clear cell RCC. **C** Coronal CT during arteriography image 63 days after biopsy shows that the biopsied mass shrinks (arrow). Its diameter is 1.7 cm, and the shrinkage rate is 26.1%

No significant difference was observed in diameters of renal mass on images before biopsy and on initial follow-up imaging (mean, 2.1 ± 0.71 cm; range, 0.8–3.8 cm; *P* = 0.90). Initial follow-up imaging showed three masses (1.6%) with > 25% shrinkage, including two clear cell RCCs and one papillary neoplasm (Fig. 5 and Table 4). One of the three masses had a reduced contrast effect on CT images.

**Table 4:** Information on three shrinking masses

	Age (y)/ Sex	Diameter (cm) before biopsy/ initial follow-up	Shrinkage rate (%)	Histological diagnosis	Procedural complication	Initial follow-up imaging	
Case						Modality	Timing
1	57/M	1.1/0.8	72.7	Clear cell RCC	Small hematoma	Dynamic CT	29 days later
2	74/W	2.3/1.7	73.9	Clear cell RCC	Small hematoma	CT during arteriography	63 days later
3	85/M	2.0/1.2	60.0	Papillary neoplasia	Small hematoma	Dynamic CT	64 days later
W: woman, M: man, RCC: renal cell carcinoma, CT: computed tomography							

## Discussion

This study showed that all 140 complications detected after the biopsy resolved or relieved, and 8 new complications (i.e., 6 small hematomas, 1 pseudoaneurysm, and 1 AVF) occurred on the initial follow-up CT or MRI. The initial follow-up image with the hematoma was performed at an earlier time, with a biopsy performed on a larger renal mass. The mean diameter of the renal masses did not significantly differ between the pre-biopsy and initial follow-up imaging; however, three masses (1.6%) shrank by more than 25% after the biopsy.

A high level of safety must be ensured for aggressive renal mass biopsies. In a systematic review of 5,228 patients, dissemination was observed in only two cases, and other complications were mainly back pain and hematoma/hematuria, most of which did not require any treatment [5]. The most common complication of a biopsy is hematoma, and its reported frequency varies (i.e., 8.5–67.9%) [8–10]. The CT was more sensitive than the US for detecting small and post-biopsy hematomas [11]. Our initial follow-up imaging showed six small new hematomas. These might not be visible on US or were negligible immediately after the biopsy and may have subsequently enlarged. Most biopsy-related hematomas are minor and asymptomatic; therefore, follow-up imaging may not usually be possible. In our study, such small hematomas spontaneously improved without treatment (hematomas were less likely to be observed over time), suggesting that strict imaging follow-up may not be necessary.

Pseudoaneurysms and AVF are rare vascular complications of renal mass biopsies. Pseudoaneurysm is generally related to renal parenchymal biopsy, nephrectomy, renal transplantation, or percutaneous procedures [12]. Renal AVFs can also result from renal parenchymal biopsy, trauma, inflammation, surgery, masses, and atherosclerosis [13]. Sosa-Barrios et al. reported that observation using color Doppler US after renal parenchymal biopsy of native kidneys showed AVF in 5.2% of patients, and 95% of them were asymptomatic [14]. The gold standard for diagnosis is angiography [15], and only one of our three AVFs could be detected on dynamic CT. In patients with renal AVF, possible symptoms include hematuria, hypertension, flank pain, vascular murmur in the renal arteries, heart failure, and decreased renal function due to hemodynamic changes [13, 14]. If patients are asymptomatic after renal mass biopsy, no follow-up imaging (e.g., color Doppler US and angiography) will be performed to diagnose the pseudoaneurysm and AVF, and it may be difficult to detect these completely on CT and MRI. All three patients with AVF were asymptomatic, and it is possible that there were other unnoticed AVFs. In our study, the frequencies of pseudoaneurysms and AVF were 1.1% and 3.3%, respectively, in patients who underwent angiography. More AVFs could have been detected if all patients had undergone angiography, because only 48.4% (92/190 patients) underwent angiography after the biopsy. In addition, it is important to perform color Doppler US for the diagnosis of renal AVF before angiography.

In our study, none of the 190 masses were enlarged (> 25%) on the initial follow-up imaging within 90 days. One active surveillance from multicenter research showed that the mean annual RCC growth rate for the entire cohort was 0.25 cm/year: masses that are ≥ 2.45 cm in the largest diameter at diagnosis grow faster than smaller masses [16]. The incidence of spontaneous regression of primary RCC is unknown; however, some English case reports have been published [17–19]. Hypothetical mechanisms for its spontaneous regression were suggested as following: humoral, immunological, and vascular factors (e.g., autoinfarction) [17]. The incidence of mass shrinkage after renal biopsy is unknown and was 1.6% (3/190 masses) in this study. One of the three masses showed a reduced contrast effect. Possible reasons for renal mass shrinkage after the biopsy include the follows: i) the mass is an inflammation-related change, not a neoplasm; ii) biopsy causes injury to feeding arteries resulting in ischemia/infarction; or iii) spontaneous regression of the mass.

This retrospective study conducted at a single institution has several limitations. First, the timing of the initial imaging follow-up was not uniform. Second, the protocol for the initial imaging follow-up was not standardized (e.g., CT or MRI or plain, contrast-enhanced, or dynamic study). Prospective follow-up with a more unified protocol may resolve these limitations; however, excessive imaging follow-up would be unnecessary because most complications are minor, asymptomatic, and improve spontaneously. Third, unenhanced CT or MRI may have overlooked pseudoaneurysms and AVFs. Finally, although 92 angiographies showed 1 pseudoaneurysm and 3 AVFs, angiography was not performed before the biopsy. Therefore, biopsy-related changes were not accurately assessed.

In conclusion, the initial follow-up imaging after the biopsy of small renal masses showed improvements in most of the complications, a few new complications, and an unchanged mass diameter.
